# Evaluation of the effect of extraction in comparison to distalization on the maxillary third molars in class II malocclusion: a retrospective study

**DOI:** 10.1007/s00784-024-05576-8

**Published:** 2024-03-04

**Authors:** Amira A. Aboalnaga, Ahmed S. Fouda

**Affiliations:** Department of Orthodontics and Dentofacial Orthopedics, Faculty of Dentistry, Cairo University, 11 EL-Saraya St. Manial, Cairo, Egypt

**Keywords:** Third molar impaction, Vertical position of third molars, Extraction, Distalization

## Abstract

**Objective:**

To compare the effects of first premolar extraction versus distalization on the vertical position and mesiodistal angulation of maxillary third molars (MxM3) in adolescent class II patients.

**Methods:**

The panoramic x-rays (OPGs) of 200 adolescent class II patients with developing MxM3s were screened. The chosen sample consisted of 2 groups: Group 1 (Distalization) comprising 48 MxM3s, and Group 2 (Extraction) comprising 50 MxM3s. The pre- and post-treatment OPGs were traced to detect the mesiodistal angulation changes of the second molars (MxM2) and MxM3s.

**Results:**

The angulation and vertical position of the MxM3s at T0 & T1 were also evaluated using Archer’s classification. The distalization group presented a non-significant decrease in the mean angulation of MxM2 and MxM3 (-2.4^o^ & -4.5^o^ uprighting respectively). In the extraction group, both MxM2 and MxM3 presented a highly significant decrease in the mean angulation (-10.5^o^ & -11^o^ uprighting respectively). The angulation and vertical position change of MxM3 significantly improved in the extraction group when compared to the distalization group (*P* < .001).

**Conclusion:**

Significant uprighting and occlusal positioning of the maxillary third molars occurred in the premolar extraction treatment group when compared to the distalization treatment group. The results of the current study highlight the importance of recognizing maxillary third molars during orthodontic treatment planning of Class II malocclusion cases.

## Introduction

Third molars (M3) are the most frequently reported impacted teeth. The global rate of M3 impaction was estimated to be about 24%, with the mandibular being more common than the maxillary M3 impaction [1]. Despite the fact that not every M3 impaction presents a clinical problem, it might still be associated with resorption of the adjacent second molar, periodontal disease, tooth decay and pathological lesions [2]. Moreover, abundant research have shown that impacted third molars seldom remain static over time [3, 4], consequently prophylactic extraction of asymptomatic impacted M3 remains a controversial topic, with some clinicians recommending early extraction to avoid aggravating the present situation [5] and others arguing that extraction can lead to potential risks and complications [6]. 

Despite some evidence suggesting that M3 impaction is controlled genetically [7], a number of studies emphasize the role of environmental factors, particularly space problems within the jaw [8]. A principle cause of maxillary third molar (MxM3) impaction is established to be the lack of retromolar space, largely relying on the maxillary tuberosity growth and the mesial drift of the upper molars [9]. Allowing or inhibiting the mesial movement of molars considerably governs the space available for MxM3 to erupt [10]. Formerly, it was delineated that at least 18 mm is required between the distal surface of the upper first molar and the Pterygoid Vertical line for proper eruption of MxM3 [11]. 

Orthodontic treatment which alter the anteroposterior position of posterior dentition ultimately influence the retromolar space available for MxM3 eruption [10, 12]. Several studies advocated the positive effect of orthodontic treatment employing premolar extraction on the eruption space and the vertical position of MxM3 [13–15]. Ǻrtun et al. [16] deduced that for every one millimeter increase in retromolar space during orthodontic treatment, the risk of MxM3 impaction is decreased by 13%. Therefore, the amount of molar mesialization taking place due to treatment is considered to be one of the most dominant predictive variables for MxM3 impaction [16] and since premolar extraction is associated with mesial movement of molars during space closure therefore it seems to diminish the frequency of third molar impaction [17]. 

Likewise, distalization of maxillary first molars is hypothesized to have a negative impact on MxM3 eruption, where hindering of the mesial movement of molars combined with the lack of tuberosity growth inevitably leads to greater chance of MxM3 impaction [10]. Both Piva et al. [18] and Miclotte et al. [19]. inferred that headgear therapy might compromise the space required for MxM3 emergence. Conversely, some evidence suggested that distalization has no significant influence on the angulation and vertical position of the MxM3 [19]. 

Therefore, this study aimed to investigate and compare the effects of distalization versus first premolar extraction orthodontic treatment on the vertical position and mesiodistal angulation of MxM3 in adolescent class II patients.

## Materials and methods

This study was approved by the Research Ethics Committee of Cairo University in Egypt. The protocol was registered at ClinicalTrials.gov. with an ID number (CEBD-CU-2021-9-7). Sample size determination performed a priori for detecting differences in MxM3 inclination at a significance level of 5% with a test power of 80%, required 43 molars in would be necessary for each group [20]. 

**Fig. 1:**
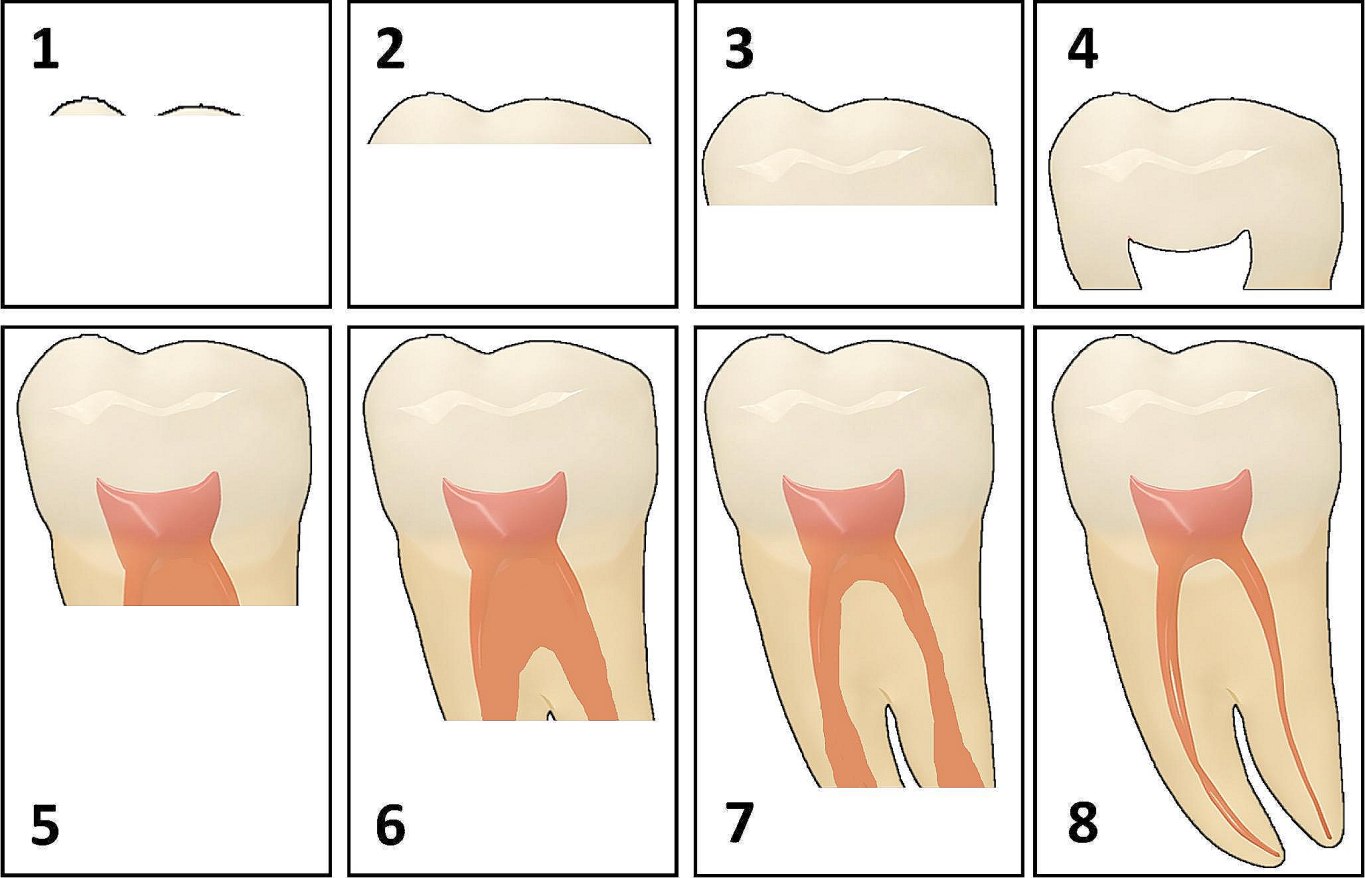
Demirjian’s classification; M3s are classified according to their developmental stage. (1) cusp tips are mineralized, (2) mineralized cusps are fused, (3) almost half of the crown is formed, (4) crown formation is complete, (5) formation of the inter-radicular bifurcation has begun and root length is less than the crown length, (6) root length is at least as great as crown length and roots have funnel-shaped endings, (7) root walls are parallel with open apices, (8) apical root ends are completely closed

The patients’ records were retrospectively selected from the Department of Orthodontics of Cairo University. The inclusion criteria were (1) Adolescent males and females with unilaterally or bilaterally developing MxM3s, (2) MxM3s were at least at developmental stage 4 according to Demirjian’s classification system [21] (Fig. 1) (3) Class II malocclusion cases that were treated by either maxillary first premolar extraction with moderate anchorage and achieved full unit class II molar relationship at the end of treatment or distalization treatment and achieved class I molar relationship at the end of treatment. The Exclusion Criteria were (1) Patients with dentofacial deformities, microdontia or hypodontia, (2) Erupted MxM3 at the beginning of treatment, (3) Cases requiring premolar extraction with maximum anchorage.

The pre- and post-treatment OPGs of 200 adolescent patients undergoing orthodontic treatment with 0.022 × 0.028-inch multibracket fixed appliances for treatment of class II malocclusion were screened. The chosen sample consisted of 2 groups: Group 1 (Distalization) comprised 26 patients (9 males & 17 females, mean age of 16.3 ± 2.9 years, 4 cases were unilateral) equivalent to 48 MxM3s, and Group 2 (Extraction) comprised 28 patients (10 males, 18 females, mean age 15.96 ± 2.46 years, 6 cases were unilateral) equivalent to 50 MxM3s. In unilateral cases, the MxM3 of the contralateral side was disregarded. All chosen Class II cases treated using distalization employed TADs for anchorage. However, the site of TADs insertion differed from one case to another, where some cases utilized buccal TADs and others used palatal TADs. Yet the net amount of distalization using different skeletal anchors is almost indistinguishable, which is about 3.5 mm on average as reported in a recent meta-analysis [22]. 

**Fig. 2:**
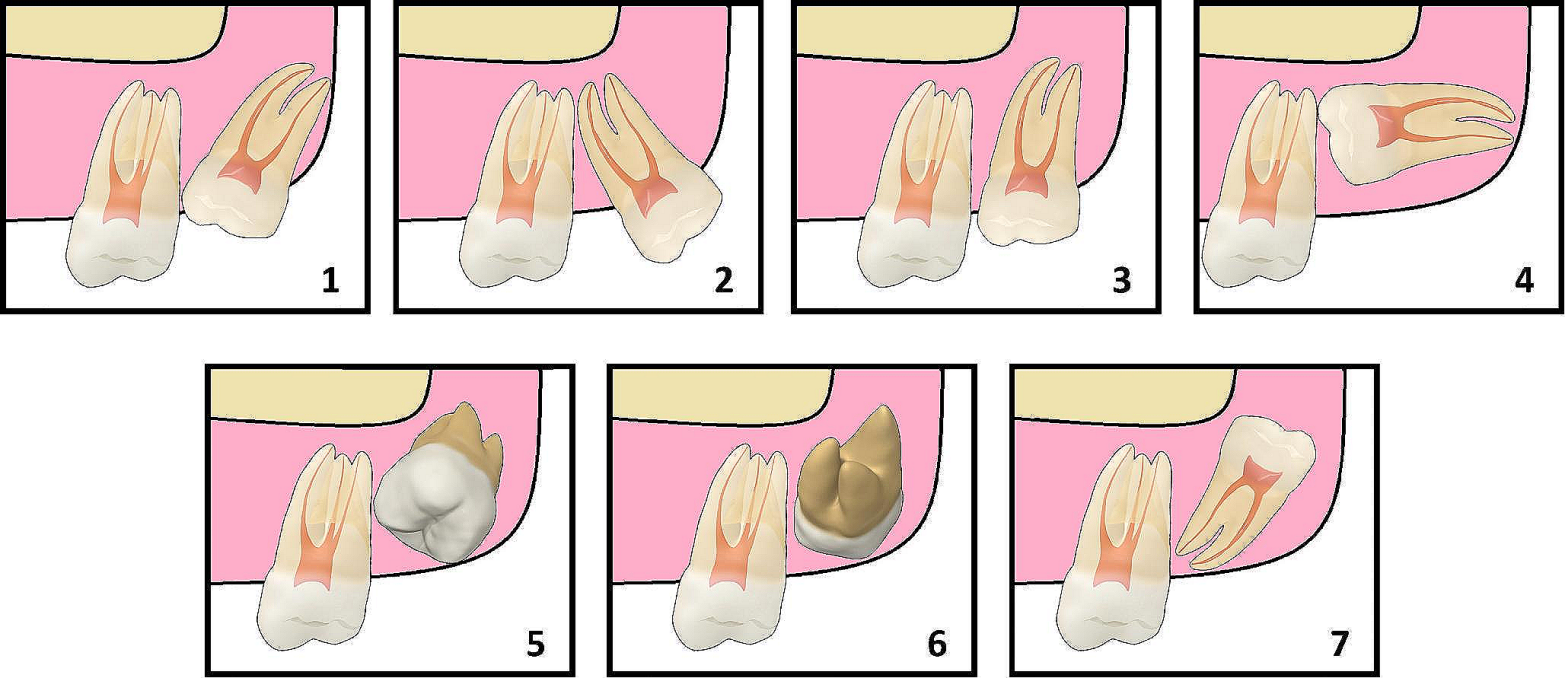
Archer’s classification of maxillary third molars according to their angulation to the long axis of the maxillary second molar. (1) mesioangular, (2) distoangular, (3) vertical, (4) horizontal, (5) buccoangular, (6) linguoangular, (7) inverted

**Fig. 3:**
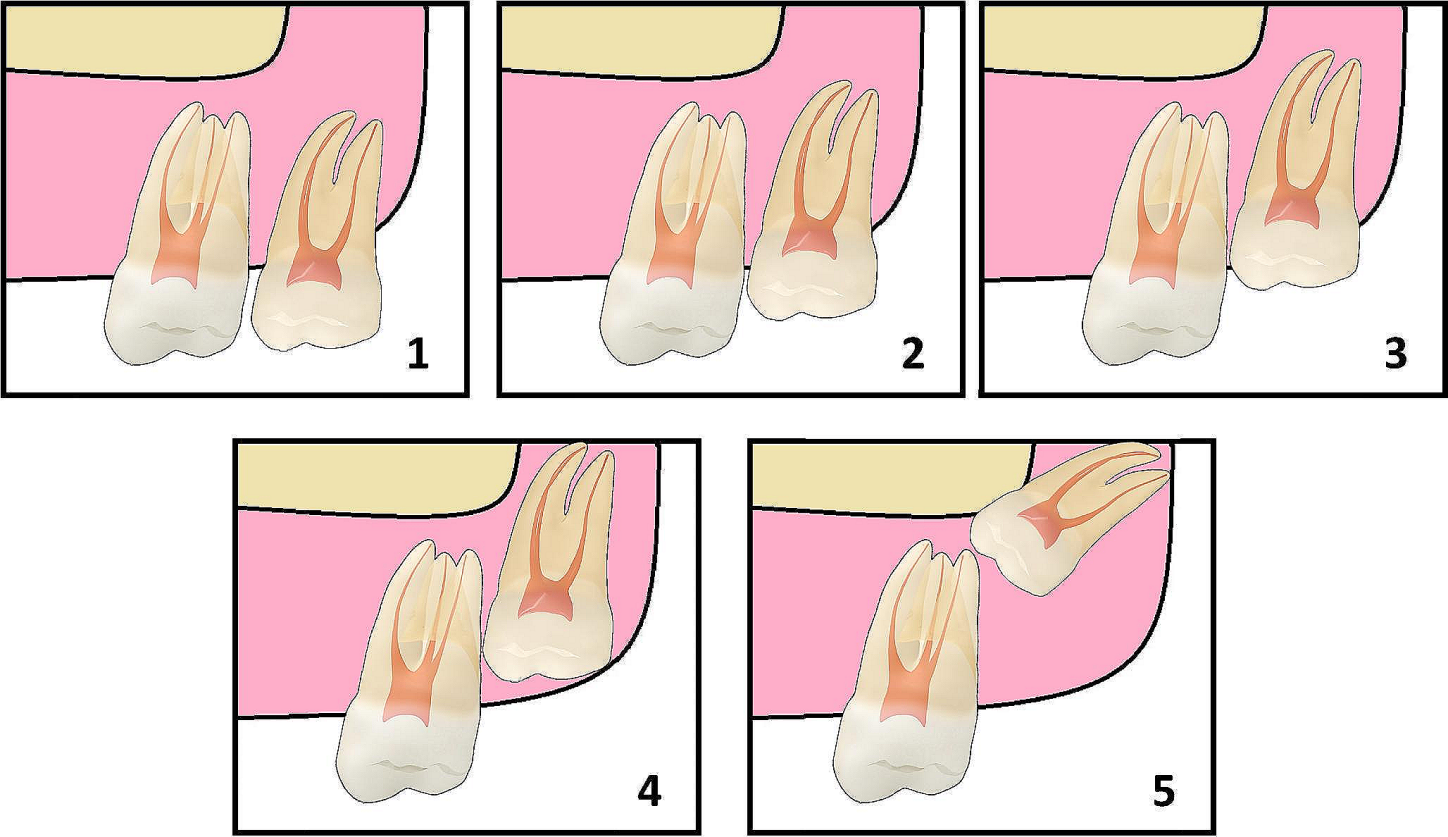
Archer’s classification of maxillary third molars according to their vertical position compared to the adjacent second molar. (1) the occlusal surface of the third molar is at the same level as the occlusal surface of the second molar, (2) occlusal surface is above the cementoenamel junction of the second molar, (3) occlusal surface is at the same level of the cementoenamel junction, (4) occlusal surface is underneath the cementoenamel junction, (5) occlusal surface is above the apex of the second molar

Pre-treatment (T0) and post-treatment (T1) OPGs were evaluated and compared for the angulation and vertical position of the MxM3s using Archer’s classification tool [23] (Figs. 2 and 3). The angulation and vertical position of each MxM3 (right and left) were evaluated separately. MxM3 angulation change was given a score from 0 to 2 where 0 = no change; when the pre-treatment and post-treatment MxM3 angulations were identical, 1 = 1 stage change; when the post-treatment angulation was one stage different than pre-treatment, or 2 = 2 stages change. In addition, each score was given a sign where a positive sign indicated angulation improvement; denoting better eruption prognosis, while a negative sign indicated angulation deterioration; denoting worse eruption prognosis.

Similarly, MxM3 vertical position change due to treatment was given a score from 0 to 3 as follows; (0 = no change; where the vertical levels of the pre-treatment and post-treatment MxM3 were identical, (1 = 1 stage change; where the post-treatment vertical position was one level different than the pre-treatment), (2 = 2 stages change), or (3 = 3 stages change). Each score was also given a sign (positive sign = vertical position improvement denoting occlusal movement), or (negative sign = vertical position deterioration denoting apical movement). Three molars in the distalization group were excluded from the evaluation using Archer’s classification tool, since the adjacent second molars were partially erupted which rendered the evaluation inaccurate.

**Fig. 4:**
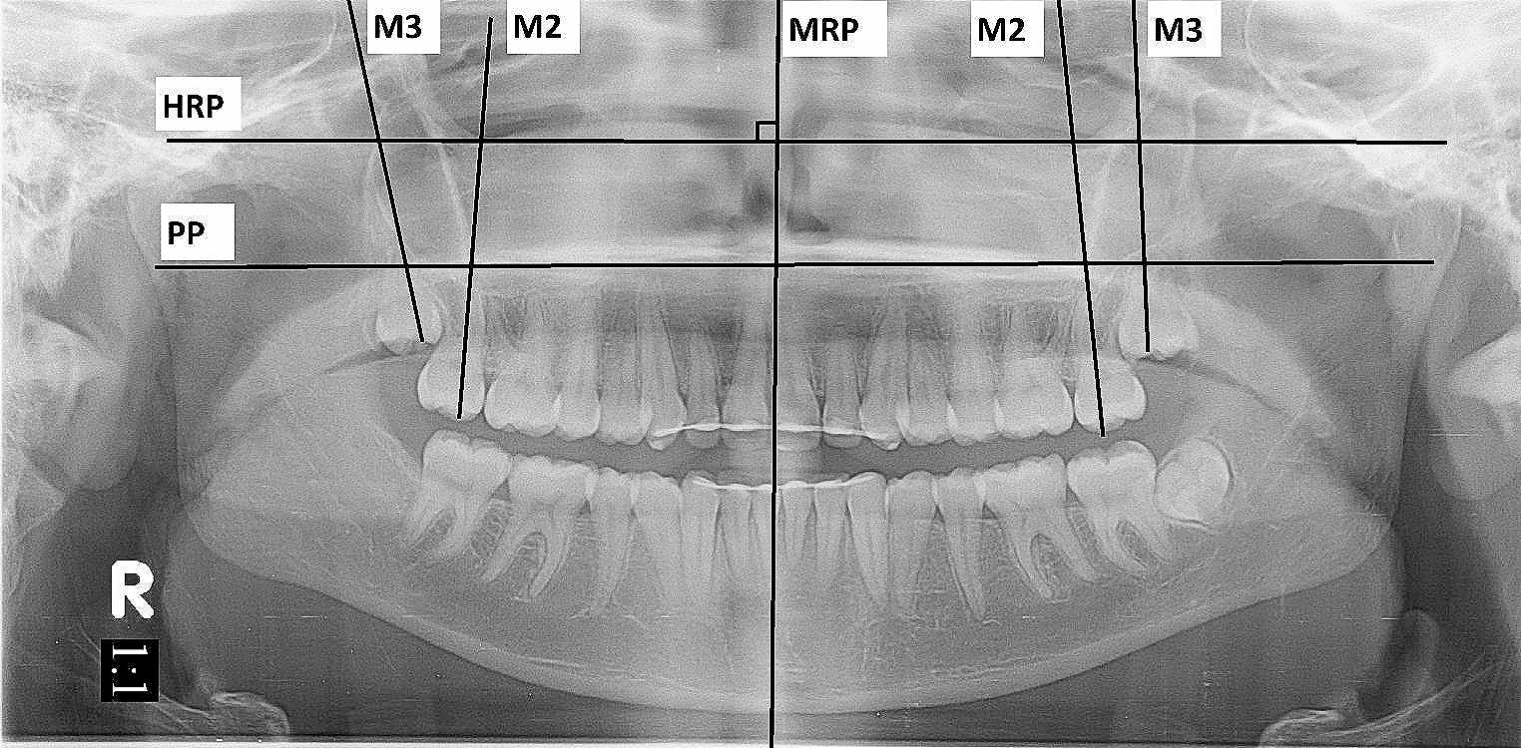
OPG showing MRP, HRP, PP and the long axes of M2 and M3 bilaterally

In addition, the OPGs were traced using facad software (IIexis AB, Linköping, Sweden) for the angular measurements. The midline reference plane (MRP) was defined as the bisector of the nasal septum and the anterior nasal spine. A horizontal reference plane (HRP) was constructed perpendicular to MRP passing through the most superior point of the nasal septum. The palatal plane (PP) was defined as a line tangent to the cranial contour of the hard palate. The long axes of MxM2 and MxM3 were the bisecting lines of the maximum mesiodistal width of each molar passing through the mesiobuccal cusp following the pulp chamber course (Fig. 4). The mesiodistal angulation of the second and third maxillary molars were measured relative to the HRP and PP at T0 & T1.

One calibrated investigator (AF) manually inserted the landmarks, and the software traced the respective lines and automatically calculated the measurements. Blinding was not possible since the investigator could distinguish the OPGs of extraction cases. 10 randomly selected radiographs were assessed twice by the same investigator and once by the second investigator (AA) to calculate the intra- and inter-observer reliability.

Mesiodistal angulation changes due to treatment were calculated as (T1 – T0). A negative value denoted an increase in molar mesioangulation (uprighting) which was considered as improvement with greater eruption possibility and vice versa.

### Statistical analysis

A standard software package (SPSS version 17.0, Chicago, Ill) was used for data analysis. Shapiro-Wilk test was used to test the normality hypothesis of variables. Only two variables were slightly deviated from normality, therefore parametric tests were utilized. Differences between T0 and T1 within the same group were evaluated using paired t-tests. Independent samples t test was used for comparing the mean difference (T1-T0) between the two groups. Descriptive statistics, including frequencies and percentages, were calculated for the categorical variables. The chi-square test was used to calculate the differences among groups for the categorical variables. In all the above statistical tools, a probability value of 0.05 was considered significant. For intra- & inter-observer reliability of all measured variables, Concordance Correlation Coefficients (CCC) were used.

## Results

All measurements showed excellent intra- and inter-observer reliability (CCC > 0.75). No significant difference was detected in the pre-treatment angulation of the second and third molars between the two groups (Table 1). The developmental staging, angulation and vertical positioning of MxM3 according to Archer classification at T0 & T1 in both groups are shown in (Tables 2 and 3). There was a significant increase detected in development during treatment (*p* < .01), however this increase did not differ between the two groups.

**Table 1:** Baseline comparison of the pre-treatment second and third molar angulations (Paired t test)

Angle (^O^)	(Group 1) Distalization group		(Group 2) Extraction group		P-value
	Mean	SD	Mean	SD	
M2/PP	105.83	8.28	107.23	8.88	0.205
M2/HRP	105.85	8.34	107.23	8.98	0.210
M3/PP	124.43	12.11	125.20	15.25	0.389
M3/HRP	124.43	12.23	125.22	15.31	0.387

M2: second molar, M3: third molar, PP: palatal plane, HRP: horizontal reference plane

*Significant (*P* < .05)

**Table 2:** Developmental staging of MxM3 according to Demirjian’s classification at T0 & T1 in both groups

Demirjian’s classification	(Group 1) Distalization		(Group 2) Extraction	
	Pre-treatment (T0)	Post-treatment (T1)	Pre-treatment (T0)	Post-treatment (T1)
Stage 4	77.78%	26.67%	42.00%	6.00%
Stage 5	20.00%	44.44%	38.00%	22.00%
Stage 6	0.00%	20.00%	6.00%	34.00%
Stage 7	2.22%	8.89%	14.00%	18.00%
Stage 8	0.00%	0.00%	0.00%	20.00%
Chi-squared	26.23		36.34	
P value	˂0.001*		˂0.001*	

*Significant (*P* < .05)

**Table 3:** The Angulation and vertical positioning of MxM3 according to Archer classification at T0 & T1 in both groups

			Pre-treatment (T0)	Post-treatment (T1)	Chi-squared	P-value
(Group 1) Distalization	MxM3 Angulation	1 (mesioangular)	0.00%	2.22%	1.16	0.560
		2 (Distoangular)	66.67%	68.89%		
		3 (vertical)	33.33%	28.89%		
		5 (Buccoangular)	0.00%	0.00%		
	MxM3 Vertical position	5 (above apex)	2.22%	2.22%	16.33	0.003*
		4 (under CEJ)	84.44%	53.33%		
		3 (at CEJ)	13.33%	28.89%		
		2 (above CEJ)	0.00%	13.33%		
		1 (at level of 7)	0.00%	2.22%		
(Group 2) Extraction	MxM3 Angulation	1 (mesioangular)	6.00%	2.00%	42.59	˂0.001*
		2 (Distoangular)	68.00%	34.00%		
		3 (vertical)	18.00%	60.00%		
		5 (Buccoangular)	8.00%	4.00%		
	MxM3 Vertical position	5 (above apex)	2.00%	0.00%	29.55	˂0.001*
		4 (under CEJ)	72.00%	26.00%		
		3 (at CEJ)	22.00%	30.00%		
		2 (above CEJ)	4.00%	24.00%		
		1 (at level of 7)	0.00%	20.00%		

*Significant (*P* < .05)

The angulation and vertical position change (T1-T0) of MxM3 significantly improved in the extraction group when compared to the distalization group (Table 4). In the distalization group, 82.2% of MxM3 showed no change in the mesiodistal angulation, 11.11% showed distal tipping and 6.67% showed mesial tipping (uprighting). As for MxM3 vertical positioning, 55.56% showed no vertical change and 45.44% showed occlusal movement. In the extraction group, 58% of MxM3 showed no change in the molar angulation and 42% showed mesial tipping (uprighting). 22% of MxM3 showed no vertical change while 78% showed occlusal movement.

**Table 4:** Comparison of the angulation and vertical position change (T1-T0) of MxM3 according to Archer’s classification between both groups

		(Group 1) Distalization	(Group 2) Extraction	Chi-squared	P-value
MxM3 Angulation change (T1-T0)	-1	11.11%	0.00%	19.26	˂0.001*
	0	82.22%	58.00%		
	+ 1	6.67%	42.00%		
MxM3 Vertical position change (T1-T0)	0	55.56%	22.00%	12.80	0.005*
	1	31.11%	44.00%		
	2	11.11%	22.00%		
	3	2.22%	12.00%		

*Significant (*P* < .05)

The distalization group showed a non-significant decrease in the mean angulation of MxM2 and MxM3 (-2.4^o^ & -4.5^o^ uprighting respectively) (Table 5). In the extraction group, both MxM2 and MxM3 presented a highly significant decrease in the mean angulation (-10.5^o^ & 11^o^ uprighting respectively) (Table 5). MxM2 and MxM3 mean angulation changes were significantly different between both groups (Table 6).

**Table 5:** Pre-treatment and post-treatment molar angulation in the two groups (Paired t test)

		Pre-treatment (T0)		Post-treatment (T1)		Change		95% CI for the change		*P*-value
		Mean	SD	Mean	SD	Mean	SD	Lower bound	Upper bound	
(Group 1) Distalization	M2/PP (°)	105.83	8.28	103.41	9.72	-2.42	12.19	-6.00	1.15	0.179
	M2/HRP (°)	105.85	8.34	103.47	9.78	-2.38	12.23	-5.97	1.21	0.189
	M3/PP (°)	124.43	12.11	119.91	17.04	-4.52	19.18	-10.15	1.11	0.113
	M3/HRP (°)	124.43	12.23	119.96	17.04	-4.47	19.20	-10.11	1.17	0.117
(Group 2) Extraction	M2/PP (°)	107.23	8.88	96.78	8.92	-10.45	10.84	-13.27	-7.62	˂0.001*
	M2/HRP (°)	107.23	8.98	96.78	9.00	-10.45	11.07	-13.34	-7.57	˂0.001*
	M3/PP (°)	125.20	15.25	114.19	16.19	-11.01	13.19	-14.51	-7.52	˂0.001*
	M3/HRP (°)	125.22	15.31	114.19	16.27	-11.04	13.17	-14.53	-7.54	˂0.001*

M2: second molar, M3: third molar, PP: palatal plane, HRP: horizontal reference plane

*Significant (*P* < .05)

**Table 6:** Comparison of the mean difference (T1-T0) of molar angulation between both groups (Independent samples t test)

	Group 1 (Distalization)		Group 2 (Extraction)		95% CI for the difference		P-value
	Mean	SD	Mean	SD	Lower bound	Upper bound	
M2/PP (°)	-2.42	12.19	-10.45	10.84	3.58	12.46	˂0.001*
M2/HRP (°)	-2.38	12.23	-10.45	11.07	3.58	12.57	˂0.001*
M3/PP (°)	-4.52	19.18	-11.01	13.19	0.18	12.81	0.044*
M3/HRP (°)	-4.47	19.20	-11.04	13.17	0.25	12.89	0.042*

M2: second molar, M3: third molar, PP: palatal plane, HRP: horizontal reference plane

*Significant (*P* < .05)

## Discussion

Maxillary third molars assume numerous degrees of distal angulation during the initial stages of development, with the mesial inclination being scarcely observed. During root development stages, uprighting ultimately takes place which is essential for eruption. Among the parameters most predictive of impaction are the mesial angulation and more than 30 degrees of distal angulation of MxM3 relative to the occlusal plane. 25% of the impactions are classified as distal due to unsatisfactory uprighting and 12% of impactions are classified as mesial due to exaggerated uprighting [16]. 

MxM3 eruption or impaction probability should be taken into consideration during orthodontic treatment planning. Up to our knowledge, this is the first study to compare the effect of distalization versus premolar extraction on MxM3 eruption prognosis, aiming to guide orthodontists in the treatment planning of such cases.

The OPG was chosen as a diagnostic tool despite the reported distortions and magnifications [24], since it’s the most commonly used x-ray for dental screening owing to the low radiation dose and feasibility. Accurately acquired OPGs were demonstrated to be a convenient tool for evaluating the mesiodistal axial inclination of teeth [25]. Angular measurements were solely utilized since linear measurements are considered inaccurate due to projection and magnification errors [14, 26]. Palatal plane was chosen to evaluate the angular changes since it’s considered more stable during growth and orthodontic treatment compared to the occlusal plane [27]. Although being reliable, the infraorbital plane was not used for evaluation in the current study since it was found to be cropped and unclear in many OPGs which will have compromised the sample size. The horizontal reference plane was used as a reliable plane to examine the reliability of the utilized palatal plane.

The selected cases were chosen to entail MxM3 at developmental stage 4 or above according to Demirjian’s classification to enable accurate tracing of the pre-treatment axial inclination. Extraction cases with maximum anchorage were excluded as per our belief that arch length preservation will negate the proposed positive impact of extraction treatment on MxM3 [28, 29]. 

As for the distalization group, the results suggest that mild angulation changes occurred (4.5^0^ uprighting) for the MxM3 which is considered clinically and statistically insignificant. Using the archer classification tool, 82% of the cases showed no change in the MxM3 mesiodistal angulation. Regarding the vertical position, a significant change was detected within the group, where almost 45% of MxM3 showed vertical improvement (occlusal movement) and 55% showed no vertical change. Surprisingly, there was no negative effect detected in the distalization group, neither distal tipping nor apical positioning of MxM3, opposing the hypothesis. A study evaluated the effect of Modified C-palatal plates distalizers on the MxM3 and reported an apical movement of 0.5 mm, however they confirmed the negligible angulation changes due to treatment [30]. Nevertheless, the long-term effects of distalization on the MxM3 position and angulation was reported to be insignificant [31, 32], which support the findings of this study. Similarly, two studies examined the effect of twinblock and forsus fatigue resistant functional appliances on the mandibular M3, and both concluded that insignificant uprighting of molars occur despite the positive influence on the retromolar space [33, 34]. 

Miclotte et al [19] reported an insignificant distal tipping (1^0^) of MxM3 due to headgear treatment. Their results indicated a possible negative effect of headgear therapy on the uprighting of MxM3. An earlier study conducted by Ghosh and Nanda [35], who explored the effect of the pendulum appliance on the maxillary molars, noted a net distal tipping of 2.5^0^ for the MxM3. The difference between the current study and the previous ones may be due to the different age groups. Previous studies were conducted on children with a mean pre-treatment age of 12 years. In the current study, the mean age was 16 years and MxM3 were chosen to be at least at the beginning of root formation stage which is normally accompanied by physiologic uprighting [16]. 

As regards the extraction group, both the vertical position and the mesiodistal angulation of MxM3 significantly improved as expected, favouring its eruption viability and supporting previous evidence [10, 12, 14, 15]. MxM3 showed an improvement in the mean mesiodistal angulation of about 11^0^ towards a more upright position.

Upon comparing the two treatment groups, the extraction treatment plan lead to significant improvement in the vertical position and axial inclination of MxM3 when compared to the distalization group. A recent study investigating the effect of extraction on maxillary third molars using CBCT reported a non-significant difference in the molar angulation due to extraction [36]. However, this was attributed to the use of skeletal anchorage in 78% of their sample.

Alfawaz et al. [37] compared the effect of total arch distalization versus maxillary premolar extraction in Class II patients. They reported no significant differences regarding all skeletal and soft tissue treatment effects except for the maxillary first molar positional changes which were highly significantly between the groups. In the distalization group, the maxillary first molars showed an average of 5.4 mm distal movement and 3.3° of distal tipping. Meanwhile in the extraction group, the first molars showed an average of 1.2 mm of mesial movement and 3.5° of mesial tipping despite the use of skeletal anchorage. Another study conducted by Chou et al. [38] examined the long-term effects of distalization treatment on the maxillary tuberosity volume in adolescent patients. They reported no significant decrease in the maxillary tuberosity volume after distalization followed by no significant increase on the long-term follow-up. In contrast, there was a highly significant increase in the maxillary tuberosity volume detected in the control group which received no treatment. It’s worth mentioning that the growth peak of the maxillary tuberosity lies between 8 and 11 years, yet it continues to grow till around the age of 20 years [39]. 

The results of the current study emphasize the importance of considering MxM3 orientation in Class II malocclusion treatment planning. It is necessary to examine the space available for MxM3 eruption before choosing the optimum treatment plan for each case. Maxillary molar distalization as a non-extraction treatment for Class II malocclusion correction should be better considered in cases with missing MxM3, or else if the sagittal space in the dental arch is sufficient. Otherwise it cannot be considered as a non-extraction treatment, since the potential need for MxM3 surgical extraction will increase.

Class II patients presented with high risk of MxM3 impaction will benefit from extraction orthodontic treatment. Meanwhile low risk cases can be treated by either distalization or premolar extraction with equivalent welfare of MxM3.

### Limitations

The observation period was restricted to the duration of active treatment, thereby it is not possible to state from the results how many third molars would have become fully erupted. Longer observation periods should be evaluated to confirm the results of this investigation. Secondly, different distalization techniques were used in the study, yet skeletal anchorage was utilized in all the cases.

## Conclusions

- Distalization has an insignificant effect on the vertical position and mesiodistal angulation of the maxillary third molars.
- Premolar extraction has a positive influence on the vertical position and mesiodistal angulation of the maxillary third molars.
- Significant uprighting and occlusal positioning of the maxillary third molars occurred in the premolar extraction group when compared to the distalization one.
